# The Role of Extracellular Vesicles in Aging and Age-Related Disorders

**DOI:** 10.3390/antiox14020177

**Published:** 2025-02-03

**Authors:** Bharathi Hassan Ganesh, Himabindu Padinjarathil, Ramya Lakshmi Rajendran, Prasanna Ramani, Prakash Gangadaran, Byeong-Cheol Ahn

**Affiliations:** 1 Dhanvanthri Laboratory, Department of Chemistry, Amrita School of Physical Sciences, Amrita Vishwa Vidyapeetham, Coimbatore 641112, India; hg_bharathi@cb.students.amrita.edu (B.H.G.); p_himabindu@cb.students.amrita.edu (H.P.); 2 Center of Excellence in Advanced Materials and Green Technologies (CoE-AMGT), Amrita School of Engineering, Amrita Vishwa Vidyapeetham, Coimbatore 641112, India; 3 Department of Nuclear Medicine, School of Medicine, Kyungpook National University, Daegu 41944, Republic of Korea; ramyag@knu.ac.kr (R.L.R.); prakashg@knu.ac.kr (P.G.); 4 BK21 FOUR KNU Convergence Educational Program of Biomedical Sciences for Creative Future Talents, Department of Biomedical Science, School of Medicine, Kyungpook National University, Daegu 41944, Republic of Korea; 5 Cardiovascular Research Institute, Kyungpook National University, Daegu 41944, Republic of Korea

**Keywords:** extracellular vesicles, intercellular communication, age-related diseases, cargo composition, therapeutic strategies

## Abstract

A variety of molecular and cellular changes distinguish the multifaceted biological process of aging. Recent studies in this decade have demonstrated the essential role of extracellular vesicles (EVs) in the aging process. Mitochondrial malfunction and increased oxidative stress are major contributors for the aging process. This review investigates the role of EVs in intercellular communication, tissue regeneration, and inflammation in the context of aging. We also discuss the exosome and its utility to reduce oxidative stress, which is a key part of aging, as well as the possibility of using the exosomes (EVs) as anti-aging drugs. Changes in cargo composition can influence the aging phenotype and impact the functionality of cells and tissues. Additionally, the role of EVs in oxidative stress during the aging process addresses potential treatment strategies and the development of biomarkers for age-associated disorders. The review also highlighted the role of exosomes in providing antioxidant properties, which help reduce excessive reactive oxygen species (ROS) and strengthen cellular defenses against oxidative stress. Additionally, it emphasized the role of extracellular vesicles (EVs) in age-related pathologies, such as neurodegenerative diseases, cardiovascular disorders, and immunosenescence, offering insights into targeted interventions for promoting healthy aging. This article provides a comprehensive analysis of the current body of knowledge regarding the therapeutic effects of EVs on aging, with a particular emphasis on the implications of this emerging field of research and its relationship to oxidative stress.

## 1. Introduction

The WHO initially released health and aging reports approximately a decade ago. It characterized ‘healthy aging’ as ‘the health-related attributes that empower individuals to engage in activities they deem valuable’ and provided an overview. The study examined the influence of biological and environmental factors on this ability [1]. Aging is characterized by a progressive and irreversible decline in the physiological activities of several bodily organs, ultimately resulting in numerous health conditions, including cardiovascular, cancerous, neurodegenerative, and musculoskeletal disorders [2]. Diverse organs and tissues in the adult body perform distinct activities and comprise various cell types. Although specialized cells, like neurons in the brain or cardiomyocytes in the heart, persist throughout an individual’s lifespan, certain cells, such as erythrocytes and intestinal epithelial cells, are replenished as necessary. Understanding the mechanism of age-related deterioration is crucial for the development of enhanced treatments [3]. Senescence is a pivotal determinant in the onset of chronic age-related disorders (AADs). Although animal models are frequently employed to investigate this interaction, they may not entirely capture the inherent complexities. Cellular aging, characterized by alterations in mechanisms including genomic instability and diminished regenerative capability, contributes to AADs. Defects in several cellular functions, including proteolytic, metabolic, and mitochondrial processes, can disturb cellular homeostasis and result in diseases [4]. AADs arise from age-related cellular deterioration that liberates deleterious chemicals into tissues, inciting inflammation and exacerbating the aging process. This pertains to the senescence-associated secretory phenotype (SASP) and impacts terminally differentiated cells such as neurons and cardiac muscle cells; such processes may result in chronic disorders, such as diabetes mellitus or neurodegenerative diseases [5]. The aging process is associated with increased oxidative stress, resulting from an imbalance between pro-oxidants and anti-oxidants, which causes molecular and cellular damage. This oxidative damage accumulates over time and may contribute to age-associated illnesses and the overall aging process. Research demonstrates a significant association between tissue concentrations of specific anti-oxidants (such as superoxide dismutase and alpha-tocopherol) and mammalian lifespan [6]. Anti-oxidants are essential in regulating autoxidation, mitigating oxidative stress, and possibly fostering healthy longevity. The relationship between anti-oxidants and aging is intricate, as some studies report inconsistent findings concerning anti-oxidant defenses in older animals. Preserving a suitable anti-oxidant/pro-oxidant equilibrium is crucial for the health of aging animals, and well-regulated anti-oxidant supplementation may enhance health in middle-aged and elderly individuals [7].

Investigations into EVs released by senescent cells are receiving increased focus, especially following the study of Valadi et al., which demonstrated capacity of EVs to transfer functional RNA to recipient cells. The EVs can be categorized based on their origin, comprising diverse proteins, lipids, and nucleic acids contingent upon the cell type [8]. Lehman et al. first described the increased excretion of EVs in senescent cells; this increase has been observed in fibroblasts, cancer cells, and epithelial cells [9]. The increased excretion of EVs occurs in cells in which senescence is activated by irradiation, oncogenic Ras expression, serial passaging, and DNA-damaging reagents [10]. Several studies are being conducted to modify EVs as a disease-modifying therapy for AADs and to assess their effects as drug-delivery vehicles for treating cancers, inflammatory disorders [11], and immune disorders. EVs are emerging as promising biological medicinal products due to their role as bio-messengers and trophic factors that influence cell behavior. Much of the preclinical work currently relies on mesenchymal stromal cells (MSCs) for sourcing EVs, utilizing established culture protocols for ex vivo expansion [12] and regenerative medicine. Nevertheless, the environment for the ex vivo expansion of MSCs must be optimized for replicating the EV isolation, production, and safety procedures in economically favorable conditions [13].

## 2. EV

EVs are a diverse population of membrane-coated vesicles released by nearly all cell types [14]. Based on their biogenesis, EVs can be classified into three primary classes: exosomes, apoptotic bodies, and shedding microvesicles [15]. EVs have been identified as key participants in cell-to-cell communication and have been linked to various physiological and pathological processes that are affected by oxidative stress. Moreover, EVs serve as long-distance intercellular communication carriers in the body and are increasingly acknowledged as potential circulating indicators of various diseases (through use in liquid biopsies) [16]. Research indicates that cells under oxidative stress secrete a greater quantity of exosomes with modified composition relative to normal circumstances [17]. Stress-induced exosomes may safeguard recipient cells from oxidative damage and facilitate angiogenesis in endothelial cells [18]. The relationship between oxidative stress and exosomes is intricate since reactive oxygen species (ROS) influence exosome release via many pathways [19]. Exosomal cargo, including mRNA, proteins, and signaling molecules, reflects the physiological condition of the originating cells and may influence the activity of destination cells. Understanding the connection between oxidative stress and exosome release is essential for elucidating their function in diverse biological processes, such as aging and pregnancy-related signaling [20].

Blood is a well-studied biofluid containing extracellular vesicles (EVs) from both blood cells and various tissues. These EVs can travel to most body tissues. Researchers are exploring the use of EV-based therapies delivered through the bloodstream due to their ability to reach different tissues directly [21]. The biological content of EVs, consisting of lipids, proteins, and nucleic acids, is most interesting. However, components of the cytoplasm or other cellular compartments can be incorporated into an EV only if they are (a) close to the budding membrane and similar in size to the EV (passive loading) or (b) specifically associated with the membrane and any energy-dependent process (active loading). Any randomly chosen molecule or cell organelle has a higher chance of being incorporated into a larger EV. Therefore, small EVs may not contain Golgi, endoplasmic reticulum, mitochondrial, or nuclear components (probably produced distantly from these places) [22]. EVs are increasingly recognized for their role in many biological processes, including aging [23]. Fresh blood rejuvenates old tissues, demonstrated using old mice injected with young EVs. Young mouse plasma small EVs (sEVs) reverse molecular, mitochondrial, cellular, and physiological aging. Research indicates that youthful sEVs increase PGC-1α expression, enhancing mitochondrial activities and reducing deficiencies in elderly tissues, partially correcting degenerative alterations and age-related malfunction [24]. Research suggests that platelets, leukocytes, and the endothelium provide most plasma-isolated EVs, with a smaller chemical repertory. These EVs change throughout aging and how these mediators affect the aging milieu needs to be uncovered. Clinical trials of plasma transfusion from young donors to Alzheimer’s patients have shown encouraging outcomes, suggesting that EVs may replicate the benefits of youthful blood [25].

**Figure 1:**
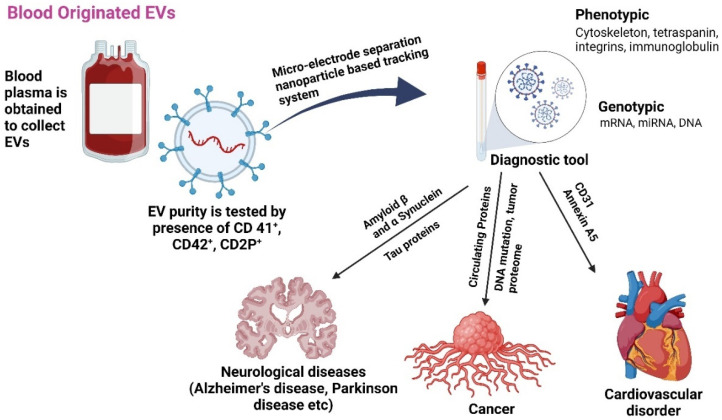
The role of EVs released from blood as biomarkers for various age-associated diseases [27,28]. Plasma-isolated exosome treatments may enhance cell bioenergetics by eliminating free radicals and act as diagnostic tools. Created with BioRender.com (accessed on 23 December 2024).

Isolating and purifying extracellular vesicles (EVs) from blood is challenging due to the complexity of separating them from plasma proteins. Despite these difficulties, the EV proteome contains valuable genetic materials, including mRNA, miRNA, and DNA, as well as proteins like immunoglobulins and integrins. High levels of glycoproteins and lipoproteins, such as cholesterol, further complicate this process. In cancer, the disease stage can be determined by analyzing proteins, mutant DNA, and tumor proteomes in EVs. Elevated levels of CD31 and annexin A5 are also noted in patients with rheumatoid and cardiovascular diseases (Figure 1) [26,27]. A study using immunomagnetic beads separated plasma CD31+ EVs from tissues related to type 2 diabetes mellitus (T2DM). The researchers identified 11 miRNAs related to vascular performance and shuttled by CD31+ EVs in a large sample of T2DM patients. Ten miRNAs were altered by T2DM, but the signature of miR-146a, -320a, -422a, and -451a successfully identified T2DM patients with problems. Another miRNA signature identified T2DM patients with a past significant adverse cardiovascular event. The study suggests that plasma CD31+ EVs’ miRNA cargo is heavily influenced by T2DM and associated problems [27].

The exact function of extracellular vesicles (EVs) in the body is not fully understood, but their presence in fluids like plasma, urine, and saliva suggests that they play an important role. Their involvement in various diseases indicates potential for use as therapeutic agents [21]. Assessment of the phenotypic and protein profiles of EVs can provide details of how well they function and whether they can be utilized as biomarkers for certain diseases. Studies on various illnesses, including Alzheimer’s disease (AD), systemic lupus erythematosus (SLE), and cancers (such as prostate, colorectal, ovarian, and non-small cell lung cancers), have revealed the potential of EVs as clinical and non-invasive biomarkers [14]. At present, EVs are thought to be a reliable and abundant source of circulating biomarkers. In the context of cancer, EVs are frequently protumorigenic; however, some host cells, such as sinus capsular macrophages in lymph nodes, can remove tumor EVs and thus act as tumor suppressors [29].

## 3. Anti-Oxidant Effect of Exosome

**Figure 2:**
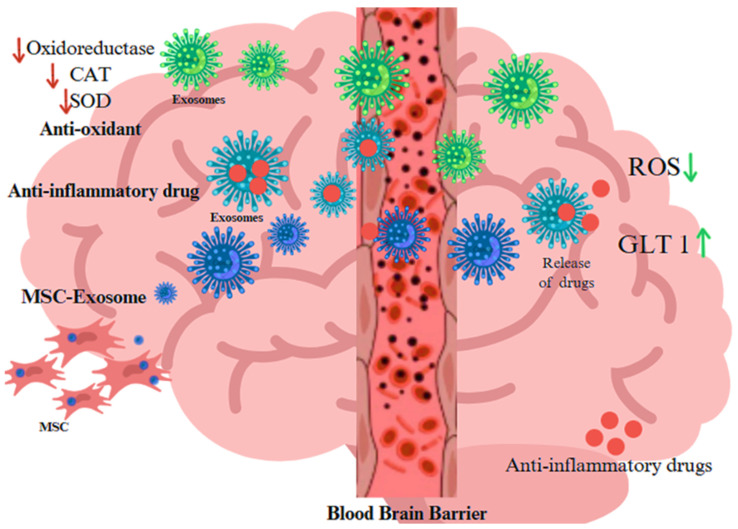
Role of EVs released from blood–brain barrier as biocarrier of anti-oxidant proteins, anti-inflammatory drugs, and exosome as derived from MSC for treatment in neurodegenerative disorders The arrow pointing indicates up, and down-regulation, whereas red increases the ageing and green suppresses the aging process Created with BioRender.com (accessed on 23 December 2024).

Oxidative stress is a significant contributor to severe and complex diseases. Exosomes can modulate this stress, potentially treating disorders by neutralizing free radicals and delivering mitochondrial proteins, thus enhancing anti-oxidant capacity. Oxidative stress is pivotal in the pathogenesis of several illnesses, including cellular damage, inflammation, and metabolic disturbances [6]. In all living cells, analogous components are accountable for facilitating excessive oxidative stress and imbalanced reduction. Consequently, exosomes may modulate these molecular components, which may be used for the treatment of many disorders. The functions of exosomes are conserved across species. Exosomes may neutralize free radicals in cells and provide mitochondrial-associated proteins, therefore directly or indirectly enhancing cellular anti-oxidant capacity and improving bioenergetics. These processes mitigate oxidative stress, demonstrating the promise of anti-oxidant effects of exosomes. Studies have shown that oxidative stress can increase exosome release from cells, including retinal pigment epithelium (RPE) cells [18]. These exosomes can carry protective messages, such as mRNAs that confer resistance to oxidative stress in recipient cells [17]. Exosomes can spread oxidatively modified molecules between cells, worsening cellular damage and aging. Protection is another exosome function along with transport anti-oxidant enzymes like SOD to scavenge ROS, reducing oxidative damage and possibly delaying aging. Anti-oxidant exosomes from MSCs reduce ROS and protect cells. Exosomes modulate oxidative stress and inflammation, making them potential therapeutic agents. MSC-derived exosomes protect retinal cells from oxidative damage, suggesting AMD treatments. Engineered exosomes can deliver therapeutic molecules to boost their anti-oxidative stress and anti-aging effects (Figure 2) [30,31]. Furthermore, exosomes released under oxidative stress conditions can promote angiogenesis in endothelial cells, potentially contributing to disease progression. These findings highlight the importance of exosomes in mediating cellular responses to oxidative stress and their potential as biomarkers and therapeutic targets in age-related diseases [18].

Reactive oxygen species (ROS) are small molecules that act as cellular messengers, regulating the proliferative response and depleting endogenous anti-oxidant reserves. They are crucial in maintaining homeostasis and can be triggered by growth factors, affecting the body’s anti-oxidative defense mechanisms. Prolonged ROS production can lead to cellular damage and diminished proliferative response. Aging is characterized by a deterioration in physiological function and heightened vulnerability to illness and mortality. Reactive species formed from COX and the transcriptional activity of interleukin-1beta (IL-1β), interleukin-6 (IL-6), tumor necrosis factor-α (TNF-α), cyclooxygenase-2 (COX-2), and inducible nitric oxide synthase (iNOS) are elevated with age. In addition to this, the nuclear factor-kappa B (NF-κB) transcription factor is recognized as the principal element in inflammation, which may be activated by oxidative stressors. The activation of NF-κB-dependent genes is a primary factor contributing to the systemic inflammatory process [32]. The free radical theory of aging-associated diseases arises from damage to cellular macromolecules caused by free radicals and the failure to adequately counteract these alterations through endogenous anti-oxidant defenses [27].

Anti-oxidants, including enzymes like glutathione peroxidase, catalase, and superoxide dismutase, play a crucial role in maintaining redox homeostasis and preventing the production of peroxides and free radicals, which can damage proteins, lipids, and DNA. This process is linked to various diseases like cancer, PD, Alzheimer’s, colitis, diabetes, liver diseases, and musculoskeletal system diseases. NADPH oxidase (PHOX) is a key protein that transfers electrons across biological membranes, producing reactive oxygen species (ROS) like H_2_O_2_, O^2−^, OH^−^, and NO·. The overexpression of PHOX can lead to excessive ROS production, causing cell damage and apoptosis. Mitochondria leakage of active oxygen is another source of ROS, as they produce ATP for subcellular processes. Oxidative phosphorylation, performed by inner mitochondrial membrane protein complexes and molecules, is essential for cellular respiration and metabolic pathways, with the help of exosomes as carriers of various signal transduction pathways occur in cells [21]. Exosomal nuclear factor erythroid 2-related factor 2 (Nrf2) modulates anti-oxidant responses and sustains cellular redox homeostasis, perhaps aiding in age-related illnesses.

## 4. Aging

Aging is a time-dependent functional deterioration that affects the cells of multicellular organisms. It is characterized by a particular set of events that impact cellular physiology, including mitochondrial failure, stem cell exhaustion, genomic instability (GI), epigenetic changes, and cellular senescence. The nine hallmarks of aging are as follows: altered intercellular communication, genomic instability, telomere attrition, epigenetic modification, loss of proteostasis, metabolic dysfunction, mitochondrial dysfunction, cellular senescence, and stem cell exhaustion. Thus, aging involves a cell-autonomous build-up of cellular and organ-specific damage to macromolecules and organelles [33,34]. Throughout an individual’s lifetime, organ and tissue functions gradually decline with aging, resulting in an increased risk of AADs and mortality. Aging is a complicated systematic process. While the effects of aging on tissue structure and function have prompted considerable research, its effects on the circulatory system are lesser known [35,36]. Cells undergo senescence as they age; this is characterized by the activation of a DNA damage response, telomere shortening, and the loss of replication potential. Aging is the most significant and common factor associated with all neurodegenerative disorders. For instance, Alzheimer’s disease (AD) is thought to affect 24 million individuals worldwide as the primary form of dementia, with individuals aged over 75 years accounting for 80% of dementia cases [37]. Cells secrete different chemicals as a result of immunosenescence and age-related alterations in other cell types. The term SASP is used to describe this condition. Therefore, EV-based therapies may offer new ways for cells to fight aging by releasing regenerative factors and reducing the number of senescent cells. Furthermore, through the release of SASP components, EVs from aged or senescent MSCs may lose their regenerative capacity and adversely affect the function of recipient cells [34]. Aging increases the risk of chronic illnesses and tissue damage. Extracellular nicotinamide phosphoribosyltransferase (eNAMPT) levels in mice and humans decrease with age. The overexpression of eNAMPT boosts NAD+ levels in tissues, improves activities, and increases female mice’s lifespan. eNAMPT is contained in EVs, which increase NAD+ production, which counteracts aging, suggesting a possible human anti-aging intervention [38].

### 4.1. Cellular Senescence

**Figure 3:**
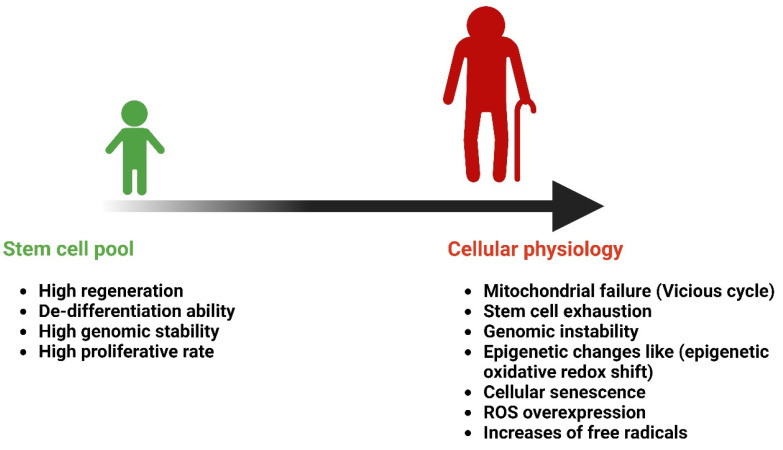
Age-related changes in cellular senescence. Created with BioRender.com (accessed on 23 December 2024).

Cellular senescence is known as a constant state of growth arrest characterized by changed cell physiology and behaviors. Senescent cells release more inflammatory cytokines and chemokines in addition to exosomes. Moreover, senescent cells exhibit a new phenotype known as the SASP. This phenotype is characterized by the release of exosomes and the secretion of many factors linked to inflammation and cancer. Senescent cells secrete cytokines. They also generate large amounts of exosomes, which have the power to alter the microenvironment when a cell is senescent (Figure 3) [24]. Oxidative stress has a crucial role in cellular senescence in the aging of tissues and organisms, and entails an irreversible cessation of growth accompanied by alterations in cell morphology, functionality, and behavior. A recent study has shown that exosomes, tiny endocytic vesicles generated by many cells for intercellular communication, contain miRNAs. Exosomes and miRNAs play a role in aging via intricate cellular senescence networks. There is significance of secretory components such as exosomes and miRNA in the regulation of cellular senescence, highlighting their possible roles in the biological processes and signaling cascades associated with senescence and aging [39]. Human aging is characterized by continuous, low-grade inflammation, called “inflammaging”. Most age-related illnesses have an inflammatory etiology, making inflammatory aging a major risk factor for senior morbidity and death. However, the exact cause of inflammaging and its possible impact on health are uncertain. To determine whether modulating inflammaging in elderly individuals is advantageous, mechanisms that affect age-related inflammation across many systems must be identified [28].

Most cell types, including tumor, dendritic, B, T, and epithelial cells, produce exosomes, which then interact with neighboring cells. Exosomes facilitate both intercellular communications by introducing regulatory substances inside the cells or receptors into the cell membranes. Exosomes are also involved in membrane trafficking and the horizontal transfer of proteins, RNAs, and miRNAs across adjacent cells, which are essential for quick phenotypic changes under various circumstances. Exosome release has been linked to senescence. A substantial increase has been reported in exosome-like microvesicles released in normal human fibroblasts during senescence [40]. Human senescent prostate cancer cells release senescence-associated exosomes, which may carry immunoregulatory and genetic information. While secreted factors and exosomes from senescent cells are linked to various biological functions, their combined impact on age-related disorders remains unclear [10,41,42].

Lehmann et al. found that therapeutically relevant radiation dosages caused early senescence as opposed to apoptosis in human prostate cancer cells [9]. Significantly more exosome-like vesicles were also secreted by these cells. The release of these vesicles was dependent on p53 activity. The involvement of p53 in EV secretion is consistent with recent findings regarding the unique role of p53 in regulating exosome release. Moreover, p53 activation regulates the transcription of tumor suppressor-activated pathway 6 (TSAP6). Specifically, Weiner-Gorzel et al. demonstrated that high miR-433-expressing cells released miR-433 into the growth media within EVs and that miR-433-mediated chemoresistance to the chemotherapeutic agent paclitaxel in ovarian cancer cells resulted from the activation of senescence genes, which are associated with exosome formation [41,43]. Moreover, miRNA caused senescence in neighboring cells and inhibited proliferation, thereby altering the tumor microenvironment. However, it has also been reported that miRNA produced within EVs inhibits recipient cell senescence [44].

One main epigenetic mechanism associated with senescence is the miRNA-mediated control of the process. Distinct miRNA signatures linked to senescence in various cell types play a role alongside extracellular vesicles (EVs), which can contain different non-coding RNAs. EVs include large EVs (l-EVs or microvesicles) and small EVs (s-EVs), differing in origin and cargo. Both types reflect the molecular characteristics of donor cells and can influence recipient cell phenotypes in systemic and paracrine ways. Increased EV secretion is considered to be a defining feature of the senescence phenotype based on data from multiple cellular models. EVs released by senescent cells exhibit proinflammatory effects that may be associated with their DNA/RNA cargo, indicating that EVs are a part of the senescent cell’s secretome. The effects of EVs on vascular aging have recently been studied. EVs derived from senescent endothelial cells and plasma of older patients reduce the rate of bone production, induce vascular calcification, and reprogram monocytes to exhibit proinflammatory traits. Moreover, EVs derived from senescent endothelial cells use their miRNA cargo to transmit pro-senescence signals to proliferating cells. Based on the finding that the replicative senescence of endothelial cells in vitro substantially mimics the progressive age-related impairment of endothelial function in vivo, we assessed the miRNAs that are differentially expressed in senescent and non-senescent human umbilical vein endothelial cells (HUVECs) and their cognate EVs (l-EVs and s-EVs) [45].

### 4.2. Genomic Instability

Genomic instability has been identified as an emerging hallmark or characteristic of most cancer types. It is defined as a process in which genomic changes increase and can influence the phenotype. EVs derived from cells are an additional route of horizontal gene transfer. The EV-mediated horizontal transfer of materials derived from tumors may cause genomic instability. RNA, proteins, and lipids can be horizontally transferred via EVs in various tumor systems, such as gliomas, monocytes, mast cells, and T cells. Moreover, tumor-derived EVs can transfer RNAs and surface elements (lipids and proteins) to monocytes [46]. Genomic instability, telomere attrition, and epigenetic changes cause cell damage, leading to normal and abnormal aging. Multiple processes, including increased DNA damage, poor repair, nuclear architectural changes, and telomere attrition, can exacerbate this damage. Epigenetic alterations in DNA, histone acetylation, methylation, chromatin-associated protein levels, and non-coding RNA activity also contribute to aging [47].

Cellular responses to DNA damage, RNA processing, and EVs associated with immune checkpoint suppression are interdependent. The RNA-processing RNA-binding proteins are essential for preserving DNA stability by controlling the transcription, mRNA splicing, and export of DNA repair proteins. In contrast, in response to DNA damage, DNA repair proteins can control the nuclear distribution of splicing factors. Together, splicing factors, RNA-binding proteins, and DNA repair proteins can resolve R-loops created during transcription and RNA processing to alleviate RNA-induced genomic instability. The increase in the effects of immune checkpoint inhibitors due to PARP inhibitors or the STING pathway is an example of the crosstalk between the immune response and cellular response to DNA damage. Tumor-derived EVs can enhance cancer spread and increase drug resistance. This can be partly attributed to PD-L1, which is released from tumor-derived EVs and functions as an RNA-binding protein to boost drug resistance in cancer cells by changing the stability of several mRNAs involved in the cellular response to DNA damage [37]. Oxidative stress in *C. elegans* leads to elevated mutation rates in mitochondrial DNA and the release of mtDNA into the cytoplasm. This can interact with cyclic guanosine monophosphate–adenosine monophosphate synthase (cGAS), resulting in the production of the second messenger cyclic GMP-AMP (cGAMP), which activates the stimulator of interferon genes (STING). This process mediates DNA damage-induced cellular senescence via STING activation and the senescence-associated secretory phenotype. DNA methylation changes with age, resulting in “epigenetic drift” and reduced acetyl-CoA production. Mitochondrial stress caused by senescence and an impaired tricarboxylic acid cycle reduce histone acetylation and alter chromatin structure [29].

**Figure 4:**
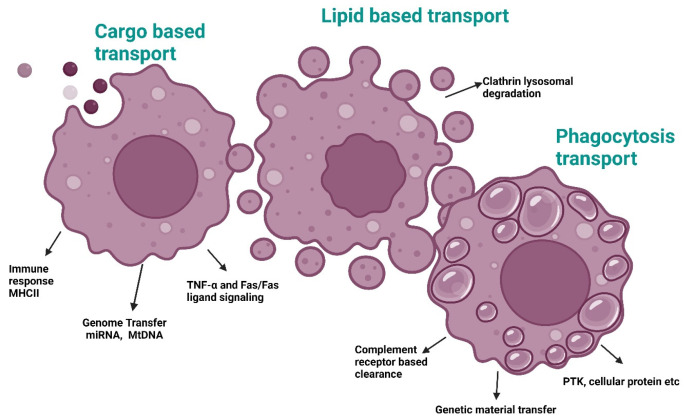
Exosome-based transport pathways involved in aging. Abbreviations: MHC II, Major histocompatibility class II; miRNA, MicroRNA; mtDNA, Mitochondrial DNA; TNF-α/Fas, Tumor necrosis factor α and Fas membrane-bound protein; PTK, Protein tyrosine kinase. Created with BioRender.com (accessed on 23 December 2024).

EVs from invading polymorphonuclear cells (PMNs) have immunomodulatory and pathogenic effects on the intestinal mucosa, especially in inflammatory bowel disease (IBD). These EVs exhibit genotoxicity, potentially leading to cancer. The release of reactive oxygen species (ROS) from PMNs contributes to oxidative stress and genotoxic effects. Recent data suggest that PMN-derived EVs may induce double-strand breaks in DNA through proinflammatory miRNAs (miR-23a and miR-155) targeting key repair regulators in intestinal epithelial cells. This results in increased genomic instability and impaired mucosal repair, hastening the development of colorectal cancer [40]. A study revealed how EV mitochondrial DNA (mt-DNA) may cause tumor formation by amplifying the radiation-induced bystander effect, in which the phenotype of irradiated cells is transferred to non-irradiated cells that are considered bystanders. That study provided evidence that EV mt-DNA from irradiated cells plays a pivotal role in inducing chromosomal instability in neighboring non-irradiated cells. This assertion was substantiated through DNase treatment and mitochondrial depletion experiments. However, the precise molecular mechanism by which EV mt-DNA induces phenotypic alterations in recipient cells remains unexplored. Furthermore, the absence of EV DNA in this model appears to be incongruent with the proposed function of EVs in eliminating damaged DNA from cells exhibiting genomic instability (Figure 4) [48].

### 4.3. Disease Spread

Exosomes and other EVs have been studied in the context of various illnesses caused by bacteria, parasites, and viruses. These studies have demonstrated alterations in the biological activity and composition of EVs. As EVs can transfer viral proteins and/or fragments of viral RNA from infected cells to target cells, their importance in viral infections has been highlighted in recent years. Notably, although the viral hijacking of EVs helps generate an environment that is favorable for viral survival by suppressing and evading the immune response, EVs can play a role in inducing antiviral responses. EVs can thus function in two ways: (a) they can promote the spread of viruses and (b) they can stimulate the immune system [49]. 

Recent studies have revealed the important roles of extracellular vesicles (EVs) in HIV pathogenesis. HIV can manipulate the endomembrane system to alter EV biogenesis, affecting target cells, cargo content, and release frequency [49]. These changes help the virus spread, reproduce, and evade the immune system. Moreover, EVs can transfer HIV co-receptors CCR5 and CXCR4 to nearby cells, increasing their susceptibility to infection and enhancing the virus’s tropism. EVs can also mask viral particles, making them less recognizable to the immune system and aiding in the dissemination of viruses to naïve cells. Additionally, EVs can help defective viral particles initiate successful infections by providing cellular proteins that assist their entry into target cells. Hepatitis C virus (HCV) similarly utilizes the vesicular pathway to assemble and release viral particles, producing vesicles that contain the entire viral genome and envelope proteins [50,51].

Extracellular vesicles (EVs) can facilitate infections by carrying viral offspring that mask the viral particles, helping them evade the immune system. EVs possess the unique ability to spread viruses or their components to uninfected cells, promoting viral transmission. While both defective and infectious viral particles are present, not all lead to successful infections due to random mutations. Vesicular transport benefits viruses by overcoming limitations; cellular proteins on EV membranes can help viral particles lacking glycoproteins enter target cells more effectively [51]. EVs can thus help defective viral particles induce a fruitful infection. Moreover, several studies have demonstrated that HCV assembles and releases viral particles via the cellular vesicular pathway; cells infected with HCV produce vesicles that include the whole viral genome, the E1 and E2 envelope proteins, or even entire viral particles [51]. EVs, like unbound viral particles, can cause productive infections once they infiltrate target cells. They offer a significant evolutionary benefit in producing viral quiescence, groups of closely similar viral genomes that constantly undergo genetic changes and compete. EV cargo may facilitate fruitful infection by genomic variants that would otherwise be negatively selected due to mutation accumulation, promoting the survival of a significant portion of viral particles [42]. Exosomes, which are membrane-derived microvesicles (30–150 nm) containing proteins (such as tetraspanins, receptor ligands, or adhesion molecules), nucleic acids, and lipids, are among the most significant components of the secretome in eukaryotic cells. The roles of EVs in a healthy physiological environment can be summarized as follows: waste management, angiogenesis, intercellular communication, cell survival, inflammation, immunological response, and coagulation. These microvesicles may contain proinflammatory cytokines, TLR4, inflammation-associated RNAs, and miRNAs, which contribute to the pathophysiology of several diseases, including cancerous, inflammatory, autoimmune, and neurodegenerative diseases [52,53]. For example, the role of exosomes in neuroinflammation has been linked to mental illnesses (such as depression, anxiety, bipolar disorder, and schizophrenia) [5]. The underlying mechanism involves a change in the release of microglial exosomes and an increase in the release of EVs derived from astrocytes and their contents in proteins related to inflammation following a toxic stimulus (such as ethanol). EVs can drive neuroinflammation by dispersing the immune response because they can cross the blood–brain barrier (BBB). The target genes of inflammatory miRNAs in brain EVs are comparable to those in plasma EVs obtained from humans and animals with alcohol abuse [54].

## 5. Therapeutic Implications of EVs in Combating AADs

### 5.1. Ocular Regeneration

**Figure 5:**
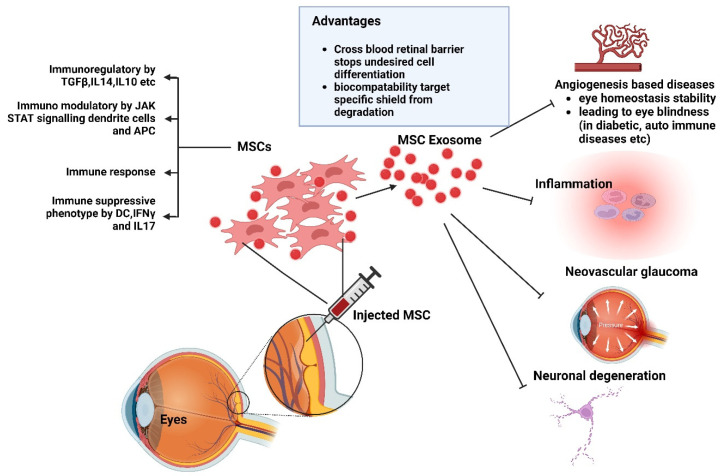
Exosomal MSC-based ocular therapy. Created with BioRender.com (accessed on 23 December 2024). Abbreviations: MSCs, Mesenchymal stomal cells; APCs, Antigen-presenting cells. Created with BioRender.com (accessed on 23 December 2024).

EVs are being researched as a possible treatment option for glaucoma, retinal degeneration, and various other ocular conditions. Research has demonstrated that EVs produced from different cell types, such as Schwann cells, MSCs, and retinal pigment epithelial (RPE) cells, can lower intraocular pressure, protect retinal cells from degeneration, and encourage corneal wound healing in glaucoma models [55]. Moreover, EVs may reduce neovascularization, neuronal degeneration, and inflammation [56]. EVs can be utilized as therapeutic carriers in ocular disease therapies, enabling patients to receive specialized treatment and directing them to specific tissues or cells for specific therapeutic compounds (Figure 5). The molecular profiles and contents of EVs offer a flexible and safe platform for administering therapeutic agents to ocular tissues. EVs also serve as fascinating markers for retinal and optic nerve damage and disorders [55].

Notably, EVs can penetrate host corneal keratocytes and ECs by passing through corneal layers [57]. The bioactive compounds therein can expedite corneal migration, proliferation, and re-epithelialization. They can also expedite the proliferation and deposition of new collagen and other extracellular matrix (ECM) proteins by stromal cells. Hence, EV therapy is a great substitute for ocular surface regeneration. Other significant mechanisms include the reduction in leukocyte infiltration, the modification of matrix metalloproteinase activity, and the reduction in apoptosis. Therapeutic compounds that rely on EVs derived from MSCs can control intercellular signaling pathways, and the created EVs can be used to treat various eye conditions [51,58]. Previous studies have assessed the role of embryonic stem cell (ESC)-derived EVs (ESC EVs) in RPE cells, confirming their cytotoxic activity using the CCK8 test [56]. After 5 days, EV-treated RPE cells showed significant differences in vitality compared to untreated and PBS-treated cells. Flow cytometry showed that EV treatment reduced the G0/G1 phase proportion while increasing the G2 phase proportion [59,60]. 

The application of ESC EVs marginally enhanced the proportion of cells in the S phase, albeit not significantly so. These findings imply that EVs successfully shield RPE cells from growth arrest. The mRNA levels of the RPE-related markers lecithin retinol acyltransferase (LRAT), retinoid isomerase (RPE65), and retinol dehydrogenase 5 (RDH5) were examined by RT-PCR to determine whether ESC EVs can enhance the function of senescent RPE cells. When senescent cells were treated with ESC EVs, the expression of RPE markers became lower in senescent cells than in young cells, suggesting that ESC EVs could improve the function of senescent RPE cells [61].

The disruption of immune privilege in the eyes can lead to autoimmune disorders like uveitis, dry eye syndrome, and optic neuritis. Exosomes can cross the blood–retinal barrier and participate in angiogenesis, which is crucial for ocular diseases like glaucoma, diabetic retinopathy, and autoimmune uveitis. Immunoregulatory factors like TGF-β, IL-4, and IL-10 can be generated by exosomes, influencing cell signal transduction and repair activity [56]. Assessment of the mechanism revealed that MSC EVs could postpone photoreceptor degeneration in Pde6b-mutant rd10 mice through the suppression of inflammation. Because of the presence of a homozygous missense mutation in the β-subunit of the rod phosphodiesterase gene Pde6b, which causes retinitis pigmentosa (RP) in humans, the rd10 mouse is a good model for studying human RP. The decrease in rod photoreceptors in the rd10 mouse model begins at postnatal day (P)17 and is almost complete by P45. Visual performance can be improved by intravenously injecting MSC EVs once a week from P14 to P28, thereby protecting deteriorated rods and cones [57].

### 5.2. Neural Regeneration in the Brain

Numerous EVs are released by astrocytes, microglia, oligodendrocytes, and neurons present in the central nervous system (CNS). EVs may serve as regulatory messengers for microglial function in the CNS. Therefore, EVs may play crucial roles in the CNS. For instance, they are involved in the synthesis of myelin sheaths, regulation of neurogenesis, and repair of injured neurons. A recent study has revealed a novel method for controlling axonal integrity. It involves the transfer of EVs from oligodendrocytes to neurons [62]. Thus, CNS-derived EVs can be isolated from systemic circulation and may be used as biomarkers for CNS diseases. The BBB is a highly selective barrier that controls transit between the peripheral nervous system and CNS. It is formed by brain microvascular cells, such as endothelial cells, astrocytes, and pericytes. Most large and tiny molecules, including medicines, recombinant proteins, and monoclonal antibodies, cannot cross the BBB, which is regarded as a barrier to the delivery of medications to the CNS [53].

EVs are relevant elements because of their notable ability to cross the BBB. Based on their cellular origin, several systems play a role in this process, which may be impacted by neuroimmune disorders. It is more plausible that EVs use the same methods as viruses to cross the BBB. Research has indicated that the ability of EVs to penetrate the BBB is partly attributed to the endocytic process. Based on existing knowledge, it is hypothesized that EVs use transcytosis to cross the BBB. In this mechanism, cells pass through vesicles in a manner similar to adsorptive transcytosis. Viruses, immune cells, and nanoparticles employ this type of mechanism to cross the BBB [40]. Besides MSC, an exosome has a significant mechanism to reduce oxidative stress by direct ROS scavenging, suppressing inflammatory proteins, inducing mitochondrial protection, and cellular biogenesis [63]. Oxidative stress is associated with neurodegeneration in Parkinson’s patients, with etiology attributed to a deficiency of natural anti-oxidants such as catalase, glutathione, and superoxide dismutase in the midbrain area. Release of inflammatory cytokines by immune cells results in neuronal death. Anti-oxidants may suppress inflammatory reactions and save dopaminergic neurons, as shown in laboratory and animal studies. Catalase, a potent anti-oxidant, has shown the ability to protect primary cerebellar granule cells in Parkinson’s disease models. Nonetheless, clinical trials have shown inadequate pharmacokinetics and a failure to traverse the blood–brain barrier [32].

The pathophysiology of Alzheimer’s disease (AD) remains unknown, with amyloid β peptides and tau playing significant roles. Exosomes (EVs) are being researched as biomarkers to predict the transition from mild cognitive impairment to overt AD and determine their role in AD pathophysiology. Alzheimer’s disease and other neurodevelopmental and neurodegenerative disorders are caused by endosomal dysfunction, particularly Down syndrome (DS) gene triplication or APOE4 expression. Brain exosomes, EVs produced and released from endosomes, have been linked to these genes. Exosome biogenesis may impact the endosomal system, and efficient exosome release may modulate neuronal flow. Greater neuronal exosome secretion may protect disease-vulnerable neurons against disruption. Therapeutic methods that maintain or promote exosome biogenesis and release may benefit various CNS illnesses [64]. The spread of AD in the brain may be caused by the build-up of altered proteins, which are toxic to neurons and transmitted across neurons by EVs. The possible impact of EVs on AD remains controversial, with both positive and negative roles reported. The release of proteins and peptides linked to AD in conjunction with exosomes is significant, and patients with AD and MCI who progress to definite AD within three years exhibit a high production of myeloid microvesicles, likely of microglial origin [48]. Exosomes play a neuroprotective role in Alzheimer’s disease (AD), capturing and clearing neurotoxic amyloid β. These exosomes, which are less glycosphingolipid-rich than glial exosomes, can suppress hazardous oligomer formation and neuronal toxicity. They can prevent synaptic plasticity-disturbing effects of synthetic and AD brain-derived amyloid β by maintaining synaptotoxic amyloid β complexes, rather than amyloid β proteolysis [53].

Furthermore, mouse primary neurons release cystatin C, a protein thought to be neuroprotective against AD, in conjunction with exosomes. An immunoproteomic study revealed at least nine distinct cystatin C glycoforms in exosomes. Moreover, all types of cystatin C (native and glycosylated) and APP metabolites within exosomes were found to decrease because of the overexpression of known AD-associated presenilin-mutant proteins. As EVs can be extracted from various body fluids and get discharged into the extracellular space, it is possible to identify proteins and other molecules linked to these vesicles for diagnostic, prognostic, and disease-monitoring purposes. EVs contain mRNA and miRNA species. Compared with non-exosomal miRNA, the analysis of enriched exosomal miRNA revealed significant improvements in the signal-to-noise ratio. This is because exosomal miRNAs are virtually diluted in circulating blood. For example, it was hypothesized that miR-193b targets the 3′-untranslated region of APP [53].

MicroRNAs like miR-132 and miR-212 are downregulated in neurodegenerative diseases like Alzheimer’s disease (AD) and frontotemporal dementia. Cheng et al. found an AD miRNA signature in exosomal miRNA from patients with AD, which could predict AD with 87% specificity and 77% sensitivity. However, the role of exosomes (EVs) in AD is not definitively established in vivo. EVs produced by tau-treated microglia and astrocytes driven by amyloid β are involved in the spread of tau and amyloid β aggregation. Further research could lead to novel approaches to diagnose and treat AD (41) [39]. According to Wang et al., synapsin, a glycoprotein linked to astrocyte EVs, may be involved in promoting axonal development and mediating neuroprotection. EVs can exhibit a neuroprotective effect [49]. The ischemia–reperfusion damage model can also exhibit neuroprotective benefits when ESCs supplemented with curcumin are used. EVs released by MSCs generated from adipose tissue can exhibit a potent neuroprotective effect on amyloid β-induced neuronal toxicity in patients with AD, reducing neuronal damage and stimulating neurogenesis [65].

In an animal model of amyotrophic lateral sclerosis (ALS), adipose-derived stem cells could reduce glial cell activation and enhance motor function. Moreover, modified EVs are useful for the treatment of diseases. Through the delivery of miR-124 into the CNS, engineered EVs can reduce cocaine-induced inflammation and microglial activation. As innovative nanotherapeutics, EVs also show great potential for treating Parkinson’s disease (PD). Moreover, EVs may serve as biomarkers for assessing severity of PD. For instance, EVs from patients with moderate and severe AD express much more myelin oligodendrocyte glycoprotein (MOG) than those from patients with mild AD. Apart from their role in neuroprotection, EVs are also involved in the pathophysiology of CNS disorders [38].

Engulfing EVs in vitro and in vivo, several CNS parenchymal cells produce them. Lachenal et al. reported the production of EVs from somatodendritic compartments of adult neurons in vitro upon glutamate activation, indicating that this is a function of regular synaptic connections. Neurons in a transwell combination system were found to release glutamate, in turn triggering oligodendrocytes to release EVs, which could be internalized by microglia and neurons. Using a similar methodology, researchers further revealed that neurons were not protected against oxidative stress and food deprivation when exosomes were extracted from an oligodendrocyte-conditioned environment. This finding suggests that oligodendrocyte-derived exosomes play a crucial role in protecting neurons [40].

Proteomic studies have demonstrated the existence of neuron-specific markers in isolated CSF EVs, such as enolase 2; transmembrane protein 132D (a marker for oligodendrocytes); vesicle-associated membrane protein 2 (VAMP2), which is a part of the exocytotic machinery of the precursor; and other microglia-specific proteins. Moreover, EVs may transport various harmful proteins, including tau, α-synuclein, or prions, which can cause and worsen neurological conditions, such as PD and AD [51]. EVs may also play a role in the clearance of excess proteins from the CNS, which may be particularly important in neurodegeneration [60].

### 5.3. Cardioprotective Effects

EVs, including microvesicles, have the potential to cure cardiovascular disorders because of their cardioprotective and therapeutic properties. This offers a viable alternative treatment option. In patients with atherosclerosis and stable coronary artery disease (CAD), neovascularization, cell migration, and inflammatory and coagulative responses are significantly mediated by EVs derived from cardiovascular cells, including cardiomyocytes, endotheliocytes, fibroblasts, platelets [52], and smooth muscle cells (SMCs). By suppressing apoptosis in patients with hyperglycemia, mesangial cell-derived EVs and cardiac parasympathetic ganglionic neurons may play a crucial role in the treatment of cardiovascular disorders. However, how secreted EVs perform their cardioprotective function or how standard cardiovascular medications alter cardiovascular cell-derived EVs remains unclear [66].

Ticagrelor, an antagonist of the P2Y12 receptor, exhibits pleiotropic effects in several clinical states in addition to receptor inhibition, as demonstrated by both in vitro and in vivo investigations. For example, Bitrium et al. reported a significant advantage of EVs produced under hypoxia-induced apoptosis in human cardiac progenitor cells (hCPCs) pretreated with ticagrelor. Furthermore, ticagrelor was found to directly impact mitochondrial dysfunction by reducing autophagosome-dependent apoptosis and inhibiting ER stress in insulin-resistant H9c2 myocytes that expressed P2Y12 receptors. The anti-oxidative and cardioprotective effects of ticagrelor-pretreated cardiomyocyte-derived EVs on hyperglycemic cardiomyocytes have been reported, taking into consideration the cardioregulatory effects of ticagrelor that contribute to EV modulation. Ticagrelor therapy can influence EVs secreted by H9c2 cells by reducing hyperglycemia, which is linked to increased ROS generation, ER stress, and autophagy. Assessment of the modulatory mechanism of ticagrelor, a popular cardiovascular medication, may enhance its beneficial effects in antiplatelet therapies [66].

MSCs are significant cells that may be extracted from several tissues, including the bone marrow. MSCs can develop into cardiac, endothelial, or vascular SMCs. According to many studies, MSCs may also function by producing EVs that have cardioprotective properties. Specifically, the activation of adenosine monophosphate-activated protein kinase (AMPK) and its downstream target, the mammalian target of rapamycin (mTOR), mediates cardioprotection. Cardioprotection also involves enhanced autophagy, resistance to apoptosis, and AKT/mTOR signaling [67]. MSCs release cardioprotective EVs after hypoxia to repeat preconditioning regimens. EV-mediated cardioprotection involves increased Cx43 expression and GSK3β inactivation via miRNA-26a regulation. EVs from mesenchymal tissue also act as inflammatory modulators, reducing cellular senescence, inflammation, and myocardial injury in patients with metabolic syndrome and renal artery stenosis. Exercise training can restore EV-mediated protection, even if protective effects are compromised due to pathological circumstances. High-intensity interval training could support the use of EVs for cardiovascular disorder prevention and treatment. Specifically, cycling exercise was observed to be linked to a notable increase in EV release; it decreased 90 min after rest. In contrast, after using a treadmill, EV release gradually decreased while being limited. An increase in EV release was most often noted in the aerobic stage of exercise. Interestingly, EVs generated following extended exercise, such as 3 weeks of swimming, may exhibit cardioprotective and antiapoptotic effects because of the stimulation of the HSP27 and ERK1/2 pathways [67].

The positive effects of consistent physical activity on CVDs can also be mediated by circulating EVs. For example, exercise can increase the levels of miR-21 inside EVs. miR-21 exhibits numerous cardioprotective effects, such as the promotion of angiogenesis through the increased expression of vascular endothelial growth factor (VEGF) and hypoxia-inducible factor-1α (HIF-1α), inhibition of apoptosis, elevation of nitric oxide synthase activity, and activation of the PTEN/AKT signaling pathway [68]. According to certain theories, in a cardiac system with Langendorff perfusion, plasma exosomes provide cardioprotection. Considering the induction of exosomes after hypoxia, their ability to transmit signals that promote cardioprotection, and their several other advantageous properties, they may facilitate the numerous distributions of the cardioprotective state through cardioprotective effects, such as remote ischemic preconditioning [68].

### 5.4. Treatment of Pulmonary Diseases

Lung-resident MSC EVs may play a role in lung tissue regeneration and repair. Experimental models of lung injury induced by bacteria, viruses, or lipopolysaccharides (LPSs) have been found to benefit from EVs derived from MSCs of various sources. In mice with ischemia–reperfusion injury, MSC EV therapy could reduce inflammation, pulmonary edema, and pulmonary dysfunction. It could also reduce pulmonary epithelial cell apoptosis induced by hypoxia or reoxygenation [69]. Exposure to irritants and allergens disrupts the coordination of the oxidant/anti-oxidant system, resulting in oxidative and nitrosative stress, which activates specific factors such as NF-κB and AP-1. The evident build-up of free radicals exposes host cells susceptible to damage by inducing apoptotic alterations [70].

There is an impact of MSC EVs on influenza virus-induced acute respiratory distress syndrome. Investigators found that MSC EVs prevented influenza virus replication [71]. The EVs also prevented the virus from inducing apoptosis in pulmonary epithelial cells of humans. Moreover, miR-21-5p in MSC EVs inhibited apoptosis induced by oxidative stress in the pulmonary epithelial cells of mice. These findings support the idea that MSC EVs in the lungs can protect against pulmonary epithelial injury, despite the lack of evidence for their role in the stem cell niche. As MSC EVs in the lungs may play a key role in delivering more specialized and focused therapies for pulmonary disorders, more research is required to fully comprehend their significance in the context of homeostasis and pulmonary disease [59].

Wang et al. demonstrated that MSC EVs could stabilize the barrier-forming function of pulmonary microvascular ECs in vitro after treatment with LPS [72]. This effect was inhibited by hepatocyte growth factor knockdown, indicating that hepatocyte growth factors may play a role in the MSC EV-mediated regulation of EC permeability [73]. EVs improve alveolar fluid clearance and enhance CD44 expression for absorption by recipient cells. Hyperoxic lung transcriptome changes and inflammation decrease after EV therapy. Alveolar macrophages absorb EVs, reducing the inflammatory state and enhancing the anti-inflammatory state. Umbilical cord MSC EVs reduce respiratory inflammation, pulmonary hypertension, and right ventricular hypertension in a neonatal mouse model [73,74,75]. 

In a model of ex vivo ischemia–reperfusion-induced acute lung injury, Gennai et al. discovered that MSC EVs enhanced alveolar fluid evacuation in human donor lungs [75]. MSC EVs have shown superior immunosuppressive and reparative properties compared to pharmaceutical treatments due to their long half-life, low immunogenicity, in vivo stability, and high delivery efficacy. They can alleviate lung damage, fasten wound closure, control endothelial and epithelial permeation, halt bacterial or viral reproduction, reduce lymphocyte penetration, and promote tissue healing [34]. Increasing research has also demonstrated that MSC EVs can influence the migration, polarization, proliferation, and maturation of distinct immune effector cells in response to the delivery of diverse transcription mediators, cytokines, and organelles. The unique immunomodulatory effects of MSC EVs can be attributed to the aforementioned properties [71,76].

EVs are secreted by lung cancer cells and are linked to several malignant characteristics in patients with cancer. According to multiple lines of evidence, EVs can mediate crosstalk between immune cells and cancer cells. Research has revealed that exosomes generated from dendritic cells prime particular cytotoxic T lymphocytes (CTLs) and initiate anticancer immune responses [77]. Exosomes generated from dendritic cells have MHC-I and MHC-II molecules on their surface, making it easier for them to directly stimulate CD41 T cells and CTLs. Moreover, increasing data indicate the potential value of dendritic cell-derived exosomes in cancer immunotherapy and for inducing tumor antigen-specific immunity [78,79].

### 5.5. Treatment of Renal and Pancreatic Damage

EVs (extracellular matrix) have shown promise in treating various diseases, including pancreatic cancer, inflammatory illnesses, chronic kidney disease (CKD), and diabetic necrosis. Exosomes obtained from MSCs have been shown to be useful in treating these conditions, as they can reduce inflammation and lower inflammatory responses [34]. In an STZ-induced type 1 diabetic animal model, exosomes from menstrual blood-derived MSCs increased β-cell mass and insulin secretion via the pancreatic and duodenal homeobox 1 (PDX-1) pathway. EVs can also improve outcomes of islet transplantation by preserving the islet architecture and increasing insulin levels in the pancreatic islets [67].

Chronic kidney disease (CKD) is a prevalent condition characterized by degenerative damage to the kidneys [68]. EVs play a crucial role in renal physiology and pathology, facilitating cell-to-cell communication and amplifying degenerative processes [69]. Research has demonstrated the therapeutic benefits of exogenous EVs, particularly those derived from MSCs, in facilitating kidney regeneration, lowering inflammation, and preventing fibrosis. Modified EVs have gained attention for improving the efficacy of EVs in treating CKD and boosting the renal tissue-targeting ability of EVs. Menopause plays a complex role in CKD development, including aging, cardiovascular disease, abnormal mineral metabolism, and increased oxidative stress due to lack of estrogen. Peroxiredoxin 3 (Prx3) is a peroxide-scavenging protein that maintains the mitochondrial redox state and anti-oxidant defense, and if local kidney lipid peroxidation is decreased, anti-oxidant defenses are relatively sustained. Lastly, increased kidney tissue lipid peroxidation, decreased anti-anti-oxidant reserves (GPx, SOD, CAT), and a changed gene expression analysis (Prx3, KIM-1, ICAM-1, COX2, TNF-α, IL-6) linked to CKD are good therapeutic agents because their complex proteins and genetic components may cure complicated ailments. Exosomes’ Prx3 can restore kidney function, decrease lipid peroxidation, and sustain anti-oxidant defenses in patients with chronic kidney disease (CKD). These exosomes, which contain complex proteins and genetic components, can treat various ailments. They can also minimize oxidative stress and sustain anti-oxidant defenses in post-menopause-chronic kidney disease [57,80]. 

**Figure 6:**
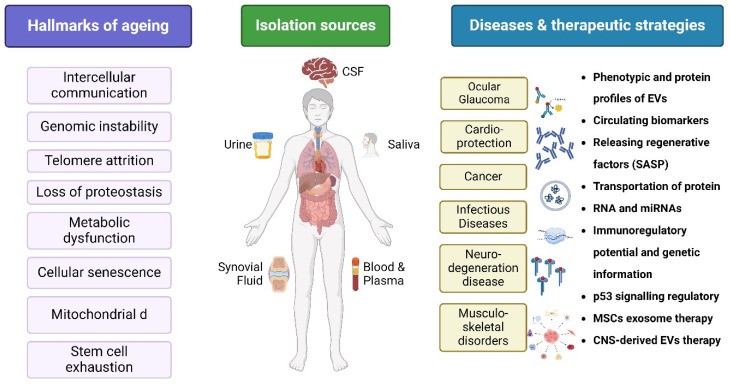
Overview on hallmarks of aging, isolation sources, diseases, and therapeutic strategies. Created with BioRender.com (accessed on 23 December 2024).

According to Tsykita et al., miR-106b-5p and miR-222-3p found in bone marrow-derived EVs may enhance β-cell proliferation through the inhibition of the calcium- and integrin-binding protein 1 (KIP) pathway [81]. A correlation between the decline in renal function and the significant decrease noted in EV production remains unclear. However, flow cytometry and specific antibodies targeting intracellular antigens have been used to identify EVs [82]. Urinary EV proteins may also be present in different types of glomerular illnesses. MSC EVs have recently gained recognition as a potentially effective treatment option for diabetic necrosis [83]. Early studies demonstrated that MSCs reduced diabetic necrosis through the paracrine impact of renal trophic factors, including EVs, in both STZ- and high-fat diet (HFD)-induced diabetic mice. EVs from human liver stem-like cells (HLSCs) and BM MSCs significantly enhanced renal function [84]. A histological study showed that renal fibrosis was considerably reduced and reversed in the group receiving EV treatment [85]. EVs include certain miRNAs that could prevent renal fibrosis in patients with diabetic necrosis by downregulating the expression of profibrotic genes [69,77]. Figure 6 highlights the core biological processes contributing to aging, EV isolation sources, diseases, and therapeutic strategies. Circulating extracellular vesicles facilitate organ communication and contribute to the exacerbation of anti-oxidant stress-based renal injury and inflammation. The molecular profile of extracellular vesicles indicates the type and pathological condition of the source cell, therefore offering promise for diagnostic and prognostic applications [86].

## 6. Conclusions

Exosomes carrying biomolecules are diminutive vesicles linked to immune system abnormalities, oncogenic situations, and neurodegenerative and autoimmune diseases. They promote the distribution of AADs by inducing oxidative stress, inflammation, and the accumulation of damaged or misfolded proteins within cells. Exosomes provide important biomarkers and prospective treatment options for autoimmune disorders. Exosomes act as clinically significant indicators for the therapeutic trajectory and response to neurological and cardiovascular therapies. Exosomes have emerged as a significant therapeutic approach for autoimmune illnesses in recent years. Nature contains nanocarriers known as exosomes, which can transport therapeutic agents to specific tissues and alter cellular signaling pathways associated with AAD. They effectively transport bioactive substances like proteins, lipids, nucleic acids, and metabolites, rendering them compelling candidates for targeted therapy. Immune cells can produce exosomes, which can be used in immunotherapy and immune regulation. Exosomes have the ability to eliminate ROS and transport mitochondrial protective proteins, thereby enhancing the anti-oxidant capacity and longevity of cells. Exosomes may mitigate oxidative stress by functioning as delivery systems for therapeutics. They can evade immune clearance through the blood–brain barrier. Exosomes demonstrate significant heterogeneity in size, internal constituents, and functionality. The internal biology and cellular microenvironment also exert influence on them. We must accurately characterize exosomes through several evaluations to fully understand their potentials. Exosomes and formulations derived from MSCs have anti-oxidant capabilities. EVs are essential for intercellular communication and are increasingly acknowledged as possible indicators and therapeutic agents in aging-related processes. The cargo of EVs may elicit both anti-oxidant and pro-oxidant responses in target cells, hence regulating redox balance in aging and age-related diseases.

## Abbreviations

EVs	Extracellular vesicles
AADs	Age-associated diseases
SASP	Senescence-associated secretory phenotype
AD	Alzheimer’s disease
SLE	Systemic lupus erythematosus
HUVECs	Human umbilical vein endothelial cells
TSAP6	Tumor suppressor-activated pathway 6
PARP	Poly ADP ribose polymerase
PD-L1	Programmed death-ligand 1
ROS	Reactive oxygen species
PMNs	Polymorphonuclear cells
CVD	Cardiovascular disease
BBB	Blood–brain barrier
RPE	Retinal pigment epithelial
LRAT	Lecithin retinol acyltransferase
RPE65	Retinoid isomerase
RDH5	Retinol dehydrogenase 5
MOG	Myelin oligodendrocyte glycoprotein
CAD	Coronary artery disease
MetS	Metabolic syndrome
VEGF	Vascular endothelial growth factor
MenSCs	Menstrual blood-derived SCs
PBMCs	Peripheral blood mononuclear cells
CKD	Chronic kidney disease
HLSCs	Human liver stem-like cells
AMPK	Adenosine monophosphate-activated protein kinase
PDX-1	Pancreatic and duodenal homeobox 1
CCL2	Chemokine ligand-2
VEGF	Vascular endothelial growth factor
ALI	Acute lung injury
IBD	Inflammatory bowel disease
ANPs	Artificial nanoparticles
TNF-α	Tumor necrosis factor alpha
PTK	Protein tyrosine kinase

## References

[1] Beard J.R., Officer A., de Carvalho I.A., Sadana R., Pot A.M., Michel J.-P., Lloyd-Sherlock P., Epping-Jordan J.E., Peeters G.M.E.E., Mahanani W.R. (2016). The World Report on Ageing and Health: A Policy Framework for Healthy Ageing. Lancet.

[2] Li Z., Zhang Z., Ren Y., Wang Y., Fang J., Yue H., Ma S., Guan F. (2021). Aging and Age-Related Diseases: From Mechanisms to Therapeutic Strategies. Biogerontology.

[3] Brunet A., Goodell M.A., Rando T.A. (2023). Ageing and Rejuvenation of Tissue Stem Cells and Their Niches. Nat. Rev. Mol. Cell Biol..

[4] Kubben N., Misteli T. (2017). Shared Molecular and Cellular Mechanisms of Premature Ageing and Ageing-Associated Diseases. Nat. Rev. Mol. Cell Biol..

[5] Ashique S., Kumar N., Mishra N., Muthu S., Rajendran R.L., Chandrasekaran B., Obeng B.F., Hong C.M., Krishnan A., Ahn B.C. (2024). Unveiling the Role of Exosomes as Cellular Messengers in Neurodegenerative Diseases and Their Potential Therapeutic Implications. Pathol. Res. Pract..

[6] Tan B.L., Norhaizan M.E., Liew W.-P.-P., Sulaiman Rahman H. (2018). Antioxidant and Oxidative Stress: A Mutual Interplay in Age-Related Diseases. Front. Pharmacol..

[7] Xiao Y., Wang S.K., Zhang Y., Rostami A., Kenkare A., Casella G., Yuan Z.Q., Li X. (2021). Role of Extracellular Vesicles in Neurodegenerative Diseases. Prog. Neurobiol..

[8] Valadi H., Ekström K., Bossios A., Sjöstrand M., Lee J.J., Lötvall J.O. (2007). Exosome-Mediated Transfer of MRNAs and MicroRNAs Is a Novel Mechanism of Genetic Exchange between Cells. Nat. Cell Biol..

[9] Lehmann B.D., Paine M.S., Brooks A.M., McCubrey J.A., Renegar R.H., Wang R., Terrian D.M. (2008). Senescence-Associated Exosome Release from Human Prostate Cancer Cells. Cancer Res..

[10] Takasugi M. (2018). Emerging Roles of Extracellular Vesicles in Cellular Senescence and Aging. Aging Cell.

[11] S Selvakumar K.V.R., S Vignesh P.R. (2018). Invitro Anti-Inflammatory Activity of Kleinia Grandiflora Leaves—Amrita Vishwa Vidyapeetham. Mater. Today Proc..

[12] Janardhanan A., Govindan S., Moorthy A., Prashanth K.V.H., Savitha Prashanth M.R., Ramani P. (2024). An Alkali-Extracted Polysaccharide from Pleurotus Eous and Exploration of Its Antioxidant and Immunomodulatory Activities. J. Food Meas. Charact..

[13] Johnson J., Wu Y.W., Blyth C., Lichtfuss G., Goubran H., Burnouf T. (2021). Prospective Therapeutic Applications of Platelet Extracellular Vesicles. Trends Biotechnol..

[14] Bæk R., Varming K., Jørgensen M.M. (2016). Does Smoking, Age or Gender Affect the Protein Phenotype of Extracellular Vesicles in Plasma?. Transfus. Apher. Sci..

[15] Prattichizzo F., Micolucci L., Cricca M., De Carolis S., Mensà E., Ceriello A., Procopio A.D., Bonafè M., Olivieri F. (2017). Exosome-Based Immunomodulation during Aging: A Nano-Perspective on Inflamm-Aging. Mech. Ageing Dev..

[16] Shao H., Im H., Castro C.M., Breakefield X., Weissleder R., Lee H. (2018). New Technologies for Analysis of Extracellular Vesicles. Chem. Rev..

[17] Eldh M., Ekström K., Valadi H., Sjöstrand M., Olsson B., Jernås M., Lötvall J. (2010). Exosomes Communicate Protective Messages during Oxidative Stress; Possible Role of Exosomal Shuttle RNA. PLoS ONE.

[18] Atienzar-Aroca S., Flores-Bellver M., Serrano-Heras G., Martinez-Gil N., Barcia J.M., Aparicio S., Perez-Cremades D., Garcia-Verdugo J.M., Diaz-Llopis M., Romero F.J. (2016). Oxidative Stress in Retinal Pigment Epithelium Cells Increases Exosome Secretion and Promotes Angiogenesis in Endothelial Cells. J. Cell Mol. Med..

[19] Zhang W., Liu R., Chen Y., Wang M., Du J. (2022). Crosstalk between Oxidative Stress and Exosomes. Oxid. Med. Cell. Longev..

[20] Jia Y.C., Ding Y.X., Mei W.T., Wang Y.T., Zheng Z., Qu Y.X., Liang K., Li J., Cao F., Li F. (2021). Extracellular Vesicles and Pancreatitis: Mechanisms, Status and Perspectives. Int. J. Biol. Sci..

[21] Alberro A., Iparraguirre L., Fernandes A., Otaegui D. (2021). Extracellular Vesicles in Blood: Sources, Effects, and Applications. Int. J. Mol. Sci..

[22] Borras C., Mas-Bargues C., Sanz-Ros J., Román-Domínguez A., Gimeno-Mallench L., Inglés M., Gambini J., Viña J. (2020). Extracellular Vesicles and Redox Modulation in Aging. Free Radic. Biol. Med..

[23] Lannigan J., Erdbruegger U. (2017). Imaging Flow Cytometry for the Characterization of Extracellular Vesicles. Methods.

[24] Chen X., Luo Y., Zhu Q., Zhang J., Huang H., Kan Y., Li D., Xu M., Liu S., Li J. (2024). Small Extracellular Vesicles from Young Plasma Reverse Age-Related Functional Declines by Improving Mitochondrial Energy Metabolism. Nat. Aging.

[25] Prattichizzo F., Giuliani A., Sabbatinelli J., Mensà E., De Nigris V., La Sala L., de Candia P., Olivieri F., Ceriello A. (2019). Extracellular Vesicles Circulating in Young Organisms Promote Healthy Longevity. J. Extracell. Vesicles.

[26] Holcar M., Kandušer M., Lenassi M. (2021). Blood Nanoparticles—Influence on Extracellular Vesicle Isolation and Characterization. Front. Pharmacol..

[27] Prattichizzo F., De Nigris V., Sabbatinelli J., Giuliani A., Castaño C., Párrizas M., Crespo I., Grimaldi A., Baranzini N., Spiga R. (2021). CD31+ Extracellular Vesicles From Patients with Type 2 Diabetes Shuttle a MiRNA Signature Associated with Cardiovascular Complications. Diabetes.

[28] Franceschi C., Campisi J. (2014). Chronic Inflammation (Inflammaging) and Its Potential Contribution to Age-Associated Diseases. J. Gerontol. Ser. A.

[29] Shao H., Chung J., Balaj L., Charest A., Bigner D.D., Carter B.S., Hochberg F.H., Breakefield X.O., Weissleder R., Lee H. (2012). Protein Typing of Circulating Microvesicles Allows Real-Time Monitoring of Glioblastoma Therapy. Nat. Med..

[30] Shao X., Zhang M., Chen Y., Sun S., Yang S., Li Q. (2023). Exosome-Mediated Delivery of Superoxide Dismutase for Anti-Aging Studies in Caenorhabditis Elegans. Int. J. Pharm..

[31] Xia C., Dai Z., Jin Y., Chen P. (2021). Emerging Antioxidant Paradigm of Mesenchymal Stem Cell-Derived Exosome Therapy. Front. Endocrinol..

[32] Haney M.J., Klyachko N.L., Zhao Y., Gupta R., Plotnikova E.G., He Z., Patel T., Piroyan A., Sokolsky M., Kabanov A.V. (2015). Exosomes as Drug Delivery Vehicles for Parkinson’s Disease Therapy. J. Control. Release.

[33] Barnham K.J., Masters C.L., Bush A.I. (2004). Neurodegenerative Diseases and Oxidative Stress. Nat. Rev. Drug Discov..

[34] Boulestreau J., Maumus M., Rozier P., Jorgensen C., Noël D. (2020). Mesenchymal Stem Cell Derived Extracellular Vesicles in Aging. Front. Cell Dev. Biol..

[35] Lazo S., Noren Hooten N., Green J., Eitan E., Mode N.A., Liu Q.R., Zonderman A.B., Ezike N., Mattson M.P., Ghosh P. (2021). Mitochondrial DNA in Extracellular Vesicles Declines with Age. Aging Cell.

[36] Alibhai F.J., Lim F., Yeganeh A., DiStefano P.V., Binesh-Marvasti T., Belfiore A., Wlodarek L., Gustafson D., Millar S., Li S.H. (2020). Cellular Senescence Contributes to Age-Dependent Changes in Circulating Extracellular Vesicle Cargo and Function. Aging Cell.

[37] Guix F.X. (2020). The Interplay between Aging-Associated Loss of Protein Homeostasis and Extracellular Vesicles in Neurodegeneration. J. Neurosci. Res..

[38] Yoshida M., Satoh A., Lin J.B., Mills K.F., Sasaki Y., Rensing N., Wong M., Apte R.S., Imai S.-I. (2019). Extracellular Vesicle-Contained ENAMPT Delays Aging and Extends Lifespan in Mice. Cell Metab..

[39] Shen L., Wang Y., Liu R., Yang Y., Liu Y., Xing B. (2023). Aging Characteristics of Degradable and Non-Biodegradable Microplastics and Their Adsorption Mechanism for Sulfonamides. Sci. Total Environ..

[40] Yin Y., Chen H., Wang Y., Zhang L., Wang X. (2021). Roles of Extracellular Vesicles in the Aging Microenvironment and Age-Related Diseases. J. Extracell. Vesicles.

[41] Choi J.H., Moon C.M., Shin T.S., Kim E.K., McDowell A., Jo M.K., Joo Y.H., Kim S.E., Jung H.K., Shim K.N. (2020). Lactobacillus Paracasei-Derived Extracellular Vesicles Attenuate the Intestinal Inflammatory Response by Augmenting the Endoplasmic Reticulum Stress Pathway. Exp. Mol. Med..

[42] Aruchamy B., Drago C., Russo V., Pitari G.M., Ramani P., Aneesh T.P., Benny S., Vishnu V.R. (2023). Imidazole-Pyridine Hybrids as Potent Anti-Cancer Agents. Eur. J. Pharm. Sci..

[43] Weiner-Gorzel K., Murphy M. (2021). Mitochondrial Dynamics, a New Therapeutic Target for Triple Negative Breast Cancer. Biochim. Biophys. Acta Rev. Cancer.

[44] Urbanelli L., Buratta S., Sagini K., Tancini B., Emiliani C. (2016). Extracellular Vesicles as New Players in Cellular Senescence. Int. J. Mol. Sci..

[45] Mensà E., Guescini M., Giuliani A., Bacalini M.G., Ramini D., Corleone G., Ferracin M., Fulgenzi G., Graciotti L., Prattichizzo F. (2020). Small Extracellular Vesicles Deliver MiR-21 and MiR-217 as pro-Senescence Effectors to Endothelial Cells. J. Extracell. Vesicles.

[46] Taylor D.D., Gercel-Taylor C. (2013). The Origin, Function, and Diagnostic Potential of RNA within Extracellular Vesicles Present in Human Biological Fluids. Front. Genet..

[47] López-Otín C., Blasco M.A., Partridge L., Serrano M., Kroemer G. (2023). Hallmarks of Aging: An Expanding Universe. Cell.

[48] Malkin E.Z., Bratman S.V. (2020). Bioactive DNA from Extracellular Vesicles and Particles. Cell Death Dis..

[49] Blandford S.N., Galloway D.A., Moore C.S. (2018). The Roles of Extracellular Vesicle MicroRNAs in the Central Nervous System. Glia.

[50] Karpman D., Tontanahal A. (2021). Extracellular Vesicles in Renal Inflammatory and Infectious Diseases. Free Radic. Biol. Med..

[51] de Toledo Martins S., Alves L.R. (2020). Extracellular Vesicles in Viral Infections: Two Sides of the Same Coin?. Front. Cell. Infect. Microbiol..

[52] Leung L., Cahill C.M. (2010). TNF-α and Neuropathic Pain—A Review. J. Neuroinflamm..

[53] Cuesta C.M., Guerri C., Ureña J., Pascual M. (2021). Role of Microbiota-Derived Extracellular Vesicles in Gut-Brain Communication. Int. J. Mol. Sci..

[54] García-Romero N., Carrión-Navarro J., Esteban-Rubio S., Lázaro-Ibáñez E., Peris-Celda M., Alonso M.M., Guzmán-De-Villoria J., Fernández-Carballal C., de Mendivil A.O., García-Duque S. (2017). DNA Sequences within Glioma-Derived Extracellular Vesicles Can Cross the Intact Blood-Brain Barrier and Be Detected in Peripheral Blood of Patients. Oncotarget.

[55] Manai F., Smedowski A., Kaarniranta K., Comincini S., Amadio M. (2024). Extracellular Vesicles in Degenerative Retinal Diseases: A New Therapeutic Paradigm. J. Control Release.

[56] Hsiao Y.P., Chen C., Lee C.M., Chen P.Y., Chung W.H., Wang Y.P., Hung Y.C., Cheng C.M., Chen C., Ko B.H. (2021). Differences in the Quantity and Composition of Extracellular Vesicles in the Aqueous Humor of Patients with Retinal Neovascular Diseases. Diagnostics.

[57] Alasmari W.A., El-Shetry E.S., Ibrahim D., ElSawy N.A., Eldoumani H., Metwally A.S., Saleh A.A., Mona M.M., Abd-Elsalam M.M., Hendam B.M. (2022). Mesenchymal Stem-Cells’ Exosomes Are Renoprotective in Postmenopausal Chronic Kidney Injury via Reducing Inflammation and Degeneration. Free Radic. Biol. Med..

[58] Liu Y., Cao X. (2016). Characteristics and Significance of the Pre-Metastatic Niche. Cancer Cell.

[59] Liu D., Liu L., Li X., Wang S., Wu G., Che X. (2024). Advancements and Challenges in Peptide-Based Cancer Vaccination: A Multidisciplinary Perspective. Vaccines.

[60] Vandendriessche C., Kapogiannis D., Vandenbroucke R.E. (2022). Biomarker and Therapeutic Potential of Peripheral Extracellular Vesicles in Alzheimer’s Disease. Adv. Drug Deliv. Rev..

[61] Liu Y., Gu S., Su Y., Wang S., Cheng Y., Sang X., Jin L., Liu Y., Li C., Liu W. (2023). Embryonic Stem Cell Extracellular Vesicles Reverse the Senescence of Retinal Pigment Epithelial Cells by the P38MAPK Pathway. Exp. Eye Res..

[62] Zheng C., Xie L., Qin H., Liu X., Chen X., Lv F., Wang L., Zhu X., Xu J. (2022). The Role of Extracellular Vesicles in Systemic Lupus Erythematosus. Front. Cell Dev. Biol..

[63] Xia C., Zeng Z., Fang B., Tao M., Gu C., Zheng L., Wang Y., Shi Y., Fang C., Mei S. (2019). Mesenchymal stem cell-derived exosomes ameliorate intervertebral disc degeneration via anti-oxidant and anti-inflammatory effects. Free. Radic. Biol. Med..

[64] Guix F.X., Ganguly A., Nassif M.C., Mathews P.M., Levy E. (2019). Exosome Production Is Key to Neuronal Endosomal Pathway Integrity in Neurodegenerative Diseases. Front. Neurosci..

[65] Chen X., Plasencia C., Hou Y., Neamati N. (2005). Synthesis and Biological Evaluation of Dimeric RGD Peptide−Paclitaxel Conjugate as a Model for Integrin-Targeted Drug Delivery. J. Med. Chem..

[66] Bitirim C.V., Ozer Z.B., Aydos D., Genc K., Demirsoy S., Akcali K.C., Turan B. (2022). Cardioprotective Effect of Extracellular Vesicles Derived from Ticagrelor-Pretreated Cardiomyocyte on Hyperglycemic Cardiomyocytes through Alleviation of Oxidative and Endoplasmic Reticulum Stress. Sci. Rep..

[67] Femminò S., Penna C., Margarita S., Comità S., Brizzi M.F., Pagliaro P. (2020). Extracellular Vesicles and Cardiovascular System: Biomarkers and Cardioprotective Effectors. Vasc. Pharmacol..

[68] Mas-Bargues C., Alique M., Barrús-Ortiz M.T., Borrás C., Rodrigues-Díez R. (2021). Exploring New Kingdoms: The Role of Extracellular Vesicles in Oxi-Inflamm-Aging Related to Cardiorenal Syndrome. Antioxidants.

[69] Kadota T., Fujita Y., Araya J., Ochiya T., Kuwano K. (2022). Extracellular Vesicle-Mediated Cellular Crosstalk in Lung Repair, Remodelling and Regeneration. Eur. Respir. Rev..

[70] Rajabi H., Konyalilar N., Erkan S., Mortazavi D., Korkunc S.K., Kayalar O., Bayram H., Rahbarghazi R. (2022). Emerging Role of Exosomes in the Pathology of Chronic Obstructive Pulmonary Diseases; Destructive and Therapeutic Properties. Stem Cell Res. Ther..

[71] Fong M.Y., Zhou W., Liu L., Alontaga A.Y., Chandra M., Ashby J., Chow A., O’Connor S.T.F., Li S., Chin A.R. (2015). Breast-Cancer-Secreted MiR-122 Reprograms Glucose Metabolism in Premetastatic Niche to Promote Metastasis. Nat. Cell Biol..

[72] Wang L., Wang N., Zhang W., Cheng X., Yan Z., Shao G., Wang X., Wang R., Fu C. (2022). Therapeutic Peptides: Current Applications and Future Directions. Signal Transduct. Target. Ther..

[73] Worthington E.N., Hagood J.S. (2020). Therapeutic Use of Extracellular Vesicles for Acute and Chronic Lung Disease. Int. J. Mol. Sci..

[74] Gangadaran P., Khan F., Rajendran R.L., Onkar A., Goenka A., Ahn B.C. (2024). Unveiling Invisible Extracellular Vesicles: Cutting-Edge Technologies for Their in Vivo Visualization. Wiley Interdiscip. Rev. Nanomed. Nanobiotechnol.

[75] Gennai S., Monsel A., Hao Q., Park J., Matthay M.A., Lee J.W. (2015). Microvesicles Derived From Human Mesenchymal Stem Cells Restore Alveolar Fluid Clearance in Human Lungs Rejected for Transplantation. Am. J. Transplant..

[76] Mohan A., Agarwal S., Clauss M., Britt N.S., Dhillon N.K. (2020). Extracellular Vesicles: Novel Communicators in Lung Diseases. Respir. Res..

[77] Jia Y., Yu L., Ma T., Xu W., Qian H., Sun Y., Shi H. (2022). Small Extracellular Vesicles Isolation and Separation: Current Techniques, Pending Questions and Clinical Applications. Theranostics.

[78] Fujita Y., Kadota T., Araya J., Ochiya T., Kuwano K. (2018). Extracellular Vesicles: New Players in Lung Immunity. Am. J. Respir. Cell Mol. Biol..

[79] Tang T.T., Wang B., Lv L.L., Dong Z., Liu B.C. (2022). Extracellular Vesicles for Renal Therapeutics: State of the Art and Future Perspective. J. Control. Release.

[80] Blanco-Colio L.M., Martín-Ventura J.L. (2023). Atherosclerosis and Other Related-Arterial Diseases. Int. J. Mol. Sci..

[81] Medeiros T., Myette R.L., Almeida J.R., Silva A.A., Burger D. (2020). Extracellular Vesicles: Cell-Derived Biomarkers of Glomerular and Tubular Injury. Cell Physiol. Biochem..

[82] Moon G.J., Sung J.H., Kim D.H., Kim E.H., Cho Y.H., Son J.P., Cha J.M., Bang O.Y. (2019). Application of Mesenchymal Stem Cell-Derived Extracellular Vesicles for Stroke: Biodistribution and MicroRNA Study. Transl. Stroke Res..

[83] Sun I.O., Lerman L.O. (2020). Urinary Extracellular Vesicles as Biomarkers of Kidney Disease: From Diagnostics to Therapeutics. Diagnostics.

[84] Gopal A., Gangadaran P., Rajendran R.L., Oh J.M., Lee H.W., Hong C.M., Kalimuthu S., Han M.H., Lee J., Ahn B.C. (2024). Extracellular Vesicle Mimetics Engineered from Mesenchymal Stem Cells and Curcumin Promote Fibrosis Regression in a Mouse Model of Thioacetamide-Induced Liver Fibrosis. Regen. Ther..

[85] Grange C., Tritta S., Tapparo M., Cedrino M., Tetta C., Camussi G., Brizzi M.F. (2019). Stem Cell-Derived Extracellular Vesicles Inhibit and Revert Fibrosis Progression in a Mouse Model of Diabetic Nephropathy. Sci. Rep..

[86] Grange C., Bussolati B. (2022). Extracellular Vesicles in Kidney Disease. Nat. Rev. Nephrol..

